# A Chaotic Particle Swarm Optimization-Based Heuristic for Market-Oriented Task-Level Scheduling in Cloud Workflow Systems

**DOI:** 10.1155/2015/718689

**Published:** 2015-08-16

**Authors:** Xuejun Li, Jia Xu, Yun Yang

**Affiliations:** ^1^Key Laboratory of ICSP, Ministry of Education, Anhui University, Hefei 230039, China; ^2^School of Computer Science and Technology, Anhui University, Hefei 230601, China

## Abstract

Cloud workflow system is a kind of platform service based on cloud computing. It facilitates the automation of workflow applications. Between cloud workflow system and its counterparts, market-oriented business model is one of the most prominent factors. The optimization of task-level scheduling in cloud workflow system is a hot topic. As the scheduling is a NP problem, Ant Colony Optimization (ACO) and Particle Swarm Optimization (PSO) have been proposed to optimize the cost. However, they have the characteristic of premature convergence in optimization process and therefore cannot effectively reduce the cost. To solve these problems, Chaotic Particle Swarm Optimization (CPSO) algorithm with chaotic sequence and adaptive inertia weight factor is applied to present the task-level scheduling. Chaotic sequence with high randomness improves the diversity of solutions, and its regularity assures a good global convergence. Adaptive inertia weight factor depends on the estimate value of cost. It makes the scheduling avoid premature convergence by properly balancing between global and local exploration. The experimental simulation shows that the cost obtained by our scheduling is always lower than the other two representative counterparts.

## 1. Introduction

Cloud computing is a pay-as-you-go model which provides resources at lower costs with greater reliability and delivers the resources by means of virtualization technologies [1]. The goal of cloud computing is to provide on-demand computing service with high reliability, scalability, and availability [2]. Workflow model is often used to manage complex scientific computing applications. A workflow is defined as a collection of tasks that are processed in a specific order [3]. And a workflow management system needs to schedule and execute the workflow efficiently to meet users' needs [4]. A cloud workflow system is a kind of platform service which facilitates the automation of workflow applications based on cloud computing. Market-oriented business model is one of the most distinguished factors between a cloud workflow system and its counterparts [5].

Workflow topology structure is very important to express relationships among tasks. Usually it is represented by task dependency graph DAG (Directed Acyclic Graph) [6]. In DAG each node indicates a workflow task and directed link represents the task dependencies. Except the root node, each node in DAG only has one parent node. By this single parent-child relationship, DAG can visually represent common workflow structures. The workflow scheduling algorithm benefits from the DAG clearly of the precedence relationships among workflow tasks.

In cloud workflow systems, hierarchical scheduling is an important and challenging issue in cloud computing facilitating [7]. The hierarchical scheduling includes two stages: service-level and task-level. The service-level scheduling deals with the assignment of tasks to services based on Quality of Service (QoS) [8]. And the cloud computing offers an entire application as a service to the end users. At task-level, the cloud computing provides kinds of on-demand Virtual Machines (VMs) to the tasks and minimizes the total cost to satisfy the QoS constraint for individual tasks. VMs that are configured before deployment have the potential to reduce inefficient resource allocation and excessive cost. In a VM there is an independently configured environment [9]. Task-level scheduling usually distributes the load on processors and maximizes their utilization. At the task-level, each scheduler manages multiple VMs. Workflow tasks and other nonworkflow tasks can be allocated to VMs [7]. Task-level scheduling can be static or dynamic. Static scheduling allocates tasks in build-time stage, while dynamic scheduling depends on system runtime states. We focus on the static scheduling in this paper. In scheduling, there are many QoS constraints, such as cost [5, 10], makespan [11], reliability [12], security [13], and availability [7]. In particular, the cost constraint is an important factor which aims to minimize the cost.

The market-oriented business model is a remarkable feature of cloud workflow systems. Many task-level scheduling strategies focus on the cost, such as communication cost, storage cost, and computation cost. In particular, the computation cost is a main part of the whole cost that we can never neglect. There are some scheduling algorithms which optimize the market-oriented scheduling cost in recent years. Workflow scheduling is a classical NP-complete problem in cloud environment [14]. Heuristic algorithms, such as Genetic Algorithm (GA) [15, 16], ACO [17, 18], and PSO [19, 20], are used to solve the task-level scheduling problems. In [15], Benedict and Vasudevan describe a GA algorithm to minimize cost in grid computing environment. In [18], Hirsch et al. present an ACO-based scheduling algorithm to optimize the makespan of scheduler within a datacenter. In particular, PSO is a typical heuristic algorithm. Netjinda et al. develop a PSO algorithm which aims at minimizing the total cost of workflow system [21]. In [22], Kumar et al. present a PSO-based heuristic algorithm to achieve the minimum cost in a cloud environment. Wu et al. propose a PSO algorithm to minimize the running cost [5]. However, the performance of GA, ACO, and PSO mostly depends on its parameters, and it has the characteristic of being trapped in local optima. In a word, these algorithms cannot achieve the optimal cost of scheduling. In this paper, we present scheduling based on Chaotic Particle Swarm Optimization (CPSO) to tackle this problem.

In cloud computing environment, it is important to consider both tasks and VMs for workflow scheduling [5]. Netjinda et al. propose an analysis of cloud workflow scheduling and a hierarchical scheduling strategy based on PSO, which is called PSO-based scheduling. As a good achievement was gained by [21], we select it as the most relevant work. This scheduling ignores the influence of premature convergence with PSO. To avoid the premature convergence, we propose the CPSO-based scheduling algorithm to reduce the cost of scheduler within a datacenter. In other word, the CPSO-based scheduling algorithm aims at minimizing the cost of the workflow system.

The rest of paper is organized as follows.  Section 2 describes a brief introduction of CPSO. In Section 3, a small workflow example is given and iteration processes of PSO and CPSO algorithms are demonstrated. In Section 4, several models including makespan, cost, and fitness for the task-level scheduling problem are built.  Section 5 presents market-oriented task-level scheduling based on CPSO.  Section 6 demonstrates experimental results.  Section 7 concludes and discusses our future work.

## 2. Overview of Chaotic PSO (CPSO)

In this section, the overview of simple PSO and chaotic PSO is given. And then chaotic sequence, fitness calculation, and adaptive inertia weight factor are introduced.

### 2.1. Simple PSO and CPSO

PSO proposed by Kennedy and Eberhart originates from exchanging and sharing of information in the process of searching for food among birds [23]. Each bird can benefit from the flight experience of another. In PSO, the particle swarm is randomly initialized to acquire initial speed and position in the feasible solution space. The track is updated through the individual optimal position and the global optimal position found by the whole swarm. Each particle constantly moves to the optimal solution and ultimately tends to the global optimal solution. However, the performance of simple PSO greatly depends on its parameters, and it is easy to achieve the local optima, which is premature convergence [13].

Therefore, much work has been carried out on the parameters modification [24], diversity increase [25], and algorithm variation [26]. To optimize PSO, the CPSO algorithm uses chaotic sequence to increase diversity [27]. Chaotic sequence can improve the diversity of solutions by high randomness and make a good global convergence by regularity. By this way, the premature convergence is avoided.

In [28], Tao et al. propose a novel CPSO-based algorithm for trustworthy workflow scheduling in a large-scale grid with a mass of service resources to optimize the scheduling performance in a multidimensional complex space. A novel CPSO algorithm is used to improve logistic map [29]. The water discharge and death penalty function are described as the decision variables. In [30], Gaing and Lin propose CPSO to solve short-term unit commitment problems with security constraints. The objective of security-constrained unit commitment is to minimize the total generation cost, which is the total of both transition cost and production cost of the scheduled units. These researches adopt chaotic sequence instead of random sequence in PSO to improve the efficiency of the algorithm.

### 2.2. Chaotic Sequence

Random sequence of PSO is very useful for simulating complex phenomena, sampling, analysis, and decision making in heuristic optimization [27]. Its quality determines the reduction of storage and computation time to achieve satisfactory accuracy. This sequence is random for one task set, but not random enough for another.

The chaos is apparently random and unpredictable and it also has an element of regularity. It is easy and fast to generate and store chaotic sequence. In CPSO, the sequence generated from chaotic systems substitute random sequence for PSO parameters. In this way, CPSO improves the global convergence and obtains a global best solution. A well-known logistic equation is donated as follows:

(1) $$\begin{array}{c} x_{n+1}=\mu \cdot x_{n}\left(\;1-x_{n}\;\right),\;0\leq x_{0}\leq 1. \end{array}$$

*μ* is the control parameter and *x* is a random variable. According to [27], *μ* is 4.

The process of the chaotic local search is defined as follows:

(2) $$\begin{array}{c} {cx}_{i}^{ITER+1}={4cx}_{i}^{ITER}\left(\;1-{cx}_{i}^{ITER}\;\right),\;i=1,2,\ldots ,n. \end{array}$$

Here, *cx*_*i*_^ITER^ is the *i*th chaotic variable with the iteration amount ITER for structure chaotic sequence. And *c* is a random number in [0,1].

### 2.3. Fitness Calculation

Fitness is to evaluate the quality of the scheduling. It is suitable for two processes: task scheduling simulation and cost calculation. Firstly, the strategy allocates the task according to the particle string. Secondly the scheduling allocates the tasks to suitable VMs and then identifies some tasks that are ready to be executed. The fitness consists of three parts. The first part is cost for the total cloud workflow scheduling. The second is penalty for scheduling when the makespan is over the deadline. The third is penalty for the idle time of VMs. In this way, fitness is an overall assessment value for the scheduling. Its calculation formula is shown in Section 4.

### 2.4. Adaptive Inertia Weight Factor

In PSO, it is critical to find a proper method to control the global and local exploration. The balance between global and local exploration is decided by the value of *w*. Obviously, the performance of PSO mostly depends on its parameters. It is clear that the influence of previous velocity is important to provide the necessary momentum for particles in the search space [31]. In order to properly control the impact of previous velocity, a suitable adaptive inertia weight factor is applied into CPSO. This weight factor depends on the optimization value of fitness calculation. The fitness value is to evaluate the quality of the solution (see Section 4). These particles with low fitness are reserved. And those particles with high fitness above the average are removed. In this way, the search space increases [32]. The adaptive inertia weight factor is described in the following formula:

(3) $$\begin{array}{c} w=\left\{\begin{array}{cc} w_{\min}+\frac{\left(w_{\max}-w_{\min}\right)\left(\text{fitness}-{\text{fitness}}_{\min}\right)}{{\text{fitness}}_{\text{avg}}-{\text{fitness}}_{\min}}, & \text{fitness}\leq {\text{fitness}}_{\text{avg}},\\ w_{\max}, & \text{fitness}>{\text{fitness}}_{\text{avg}}. \end{array}\right. \end{array}$$

Here, *w*_min⁡_ and *w*_max⁡_ are the maximum and minimum of *w*. fitness_min⁡_ and fitness_avg_ donate the minimum and average fitness of all particles.

Obviously, larger inertia weight factor leads particles to global search, whilst smaller factor guides particles to current local search. Thus, a proper factor is significant to find the best possible solution accurately and efficiently. In other words, the adaptive inertia weight factor provides a good way to preserve diversity of population and maintain good convergence.

## 3. Problem Analysis

At first, a small example of workflow is given. Secondly, in order to clearly display how the performance of CPSO is better than that of PSO, the scheduling plan iteration processes of PSO and CPSO algorithms are demonstrated in detail. In addition, time and cost of scheduling plan are compared.

### 3.1. Small Example of Workflow

**Figure 1:**
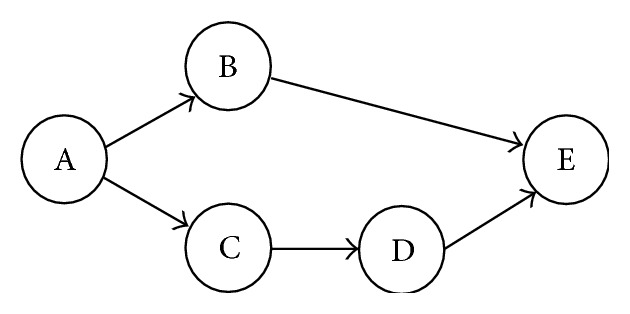
Small example of workflow.

An example of workflow by task dependency graph DAG is given in Figure 1. After task A has executed, tasks B, C are ready to execute. Task D will execute after task C. When tasks B, D have finished, task E is ready. The execution time of task on VM type is shown in Table 1.

### 3.2. Iteration Processes of PSO and CPSO Algorithms

The PSO algorithm is divided into four processes: scheduling plan initialization, update, cost calculation, and selection. Different from PSO, the CPSO algorithm uses chaotic sequence in scheduling plan initialization and update.

Table 2 shows iteration processes of PSO and CPSO algorithms. The VM types of small, medium, and large are represented by 1, 2, and 3, respectively. *x*_*m*_^*n*^ and *v*_*m*_^*n*^ represent the position and velocity of the *m*th particle in the *n*th iteration. *x*_*m*_^*n*^ and *v*_*m*_^*n*^ initialize randomly from 0 to 1 in PSO, while they do by chaotic sequence in CPSO. Plan_*i*_^*j*^ is scheduling plan of the *i*th particle in the *j*th iteration. Its value is VM type (1–3). Best_*k*_ is the best scheduling plan in the *k*th iteration. ET represents the execution time of scheduling plan.

The scheduling plan iteration continues until it reaches the maximum number of iterations. As shown in Table 2, there are 3 iterations in both PSO and CPSO. The best scheduling plan of the last iteration is the final solution. It is clear that the time and cost of final CPSO solution are lower than PSO. The CPSO algorithm finds better scheduling plan.

### 3.3. Comparison of Scheduling Plan

**Figure 2:**
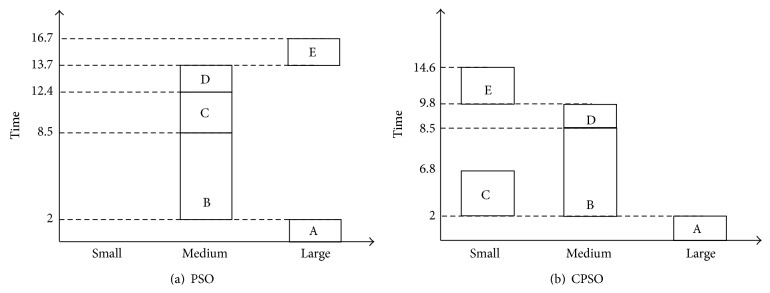
Gantt charts of scheduling plans.

After iteration processes of PSO and CPSO algorithms, the scheduling plans are generated and shown by Gantt chart in Figure 2. These charts are clear to express scheduling plans.

The per-hour cost for small, medium, and large instance is 0.12, 0.24, and 0.48, respectively. From the Gantt chart in Figure 2, the total execution time of scheduling plan generated by PSO is 16.7 and the total cost is 4.3, while the total execution time of scheduling plan generated by CPSO is 14.6 and the total cost is 3.98. Therefore, time and cost of CPSO's scheduling plan are less than those of PSO. It can be drawn that performance of CPSO is better than PSO.

## 4. Models for Task-Level Scheduling Problem

In this section, firstly several basic definitions are given. Then some models including makespan, cost, and fitness for task-level scheduling optimization problem are presented.

**Definition 1 .**

Task *T*_*i*_ is donated as 〈time, cost〉, where time is the execution time of *T*_*i*_ and cost is the expense.

**Definition 2 .**

VM *V*_*j*_ is defined as 〈price, speed〉, where price is the purchase price of  *V*_*j*_ and speed is the execution speed set according to Amazon EC2 (http://aws.amazon.com/cn/ec2/).

**Definition 3 .**

Workflow *W* = (*T*_*i*_, *V*_*j*_) is denoted as a DAG, where *T*_*i*_ is a task and *V*_*j*_ is a VM.

**Definition 4 .**

*S*_*k*_ = {(*T*_*i*_1_,_ *V*_*j*_1__),…, (*T*_*i*_*n*_,_ *V*_*j*_*m*__)} is the scheduling of tasks on VMs, where (*T*_*i*_*n*_,_ *V*_*j*_*m*__) is task *T*_*i*_*n*__ assigned to VM *V*_*j*_*m*__.

**Definition 5 .**

Task set TS_*j*_ which includes some tasks on *V*_*j*_ is represented by 〈cost, makespan, wastetime〉. cost is the total cost of TS_*j*_, makespan is the total execution time, and wastetime is the idle time; TS_*j*_^*S*_*k*_^ · wastetime = TS_*j*_^*S*_*k*_^ · makespan − ∑_*i*=1_^*n*^ *T*_*i*_^*j*^ · time. Here, TS_*j*_^*S*_*k*_^ · makespan is the execution time of TS_*j*_ with *S*_*k*_ and *T*_*i*_^*j*^ · time is the execution time of *T*_*i*_ on *V*_*j*_.

According to Definitions 1–5, three models makespan, cost, and fitness are built by Formulas (4)–(11) as follows.

Firstly Makespan_*S*_*k*__ is the maximum makespan of all tasks with *S*_*k*_:

(4) $$\begin{array}{c} {\text{Makespan}}_{S_{k}}=\underset{1\leq j\leq m}{\max}\left\{\;{\text{TS}}_{j}^{S_{k}}\cdot \text{makespan}\;\right\}. \end{array}$$

Here, TS_*j*_^*S*_*k*_^ · makespan is the total of the vacant time and execution time of TS_*j*_:

(5) $$\begin{array}{c} {\text{TS}}_{j}^{S_{k}}\cdot \text{makespan}={\text{EFT}}_{i}-{\text{EST}}_{i}. \end{array}$$

EST_*i*_ is the earliest start time of TS_*j*_:

(6) $$\begin{array}{c} {\text{EST}}_{i}=\max\left(\;{\text{Finish}}_{i-1},{\text{Available}}_{j}\;\right). \end{array}$$

Here, Finish_*i*−1_ is the completion time of the preceding task of *T*_*i*_ and Available_*j*_ is the available time of *V*_*j*_.

EFT_*i*_ is the earliest finish time of *T*_*i*_ and its succeeding tasks:

(7) $$\begin{array}{c} {\text{EFT}}_{i}={\text{Start}}_{i}+{\text{RT}}_{i}. \end{array}$$

Here, RT_*i*_ is the total of the execution time of *T*_*i*_ and all of its succeeding tasks and Start_*i*_ is the start time of *T*_*i*_:

(8) $$\begin{array}{c} {\text{RT}}_{i}=T_{i}^{j}\cdot \text{time}+\max\left(\;{\text{RT}}_{k}\;\right). \end{array}$$

Here, RT_*k*_ is the execution time of succeeding tasks.

Deadline is the upper limit of the makespan. deadline_min⁡_ is the minimum deadline, and deadline_max⁡_ is the maximum deadline.

(9) deadlinemin⁡=min⁡MakespanSk,deadlinemax⁡=max⁡MakespanSk.

Secondly Cost_*S*_*k*__ is the total cost of *S*_*k*_. It evaluates the performance of *S*_*k*_. The less the cost of scheduling plan *S*_*k*_, the better the performance of this scheduling. Consider

(10) $$\begin{array}{c} {\text{Cost}}_{S_{k}}=\overset{m}{\underset{j=1}{\sum }}{\text{TS}}_{j}^{S_{k}}\cdot \text{cost}. \end{array}$$

Here, TS_*j*_^*S*_*k*_^ · cost = *V*_*j*_ · price × TS_*j*_^*S*_*k*_^ · makespan is the cost of a task set TS_*j*_, and *V*_*j*_ · price is the purchase price of *V*_*j*_. TS_*j*_^*S*_*k*_^ · makespan is the total of the vacant time and execution time of TS_*j*_. The cost calculation is divided into two parts: price of VM and the total makespan of tasks. At the same price of VM condition, the more the makespan of tasks, the higher the cost of this scheduling plan.

At last, fitness evaluates the quality of the scheduling and is shown in the following formula according to [21]:

(11) fitness=t1×CostSk+t2×10×CostSk×MakespanSkdeadline+∑i=1mVj·price×TSjSk·wastetime.

Here, if Makespan_*S*_*k*__ does not exceed the given deadline, *t*_1_ is 1 and *t*_2_ is 0; otherwise, *t*_1_ is 0 and *t*_2_ is 1. Cost_*S*_*k*__, Makespan_*S*_*k*__, and deadline can be calculated by Formulas (4) and (9). Here, TS_*j*_^*S*_*k*_^ · wastetime is the idle time of *V*_*j*_ with *S*_*k*_.

The execution time of task set is decided by the maximum makespan of tasks (Formula (4)). The makespan of task set is calculated by EST, RT, and EFT (Formulas (6)–(8)). The deadline is the upper limit of the makespan (Formula (9)). The cost of scheduling plan (Formula (10)) depends upon the impact of three factors: cost and performance of VM (Definition 2), execution time of task set (Definition 5), and scheduling plan of tasks (Definition 4). The fitness is an overall assessment value for the scheduling (Formula (11)). Obviously, when the value of fitness is smaller, the cost is less and then the scheduling is more efficient. Otherwise, it is inefficient.

## 5. Market-Oriented Task-Level Scheduling Based on CPSO

To avoid the premature convergence, we propose a novel market-oriented scheduling algorithm based on CPSO to reduce the cost within a datacenter. This algorithm is divided into two parts. From lines (3) to (6), scheduling plan is initialized and fitness is updated. From lines (8) to (15), the scheduling strategy allocates the tasks to suitable VMs. Chaotic sequence improves the diversity of solutions by high randomness and assures a good global convergence by regularity. The fitness evaluates the quality of the scheduling. And adaptive inertia weight factor depends on fitness. Because it is a proper balance between global and local exploration, it makes the scheduling avoid premature convergence.

**Algorithm 6 (market-oriented task-level scheduling).**

The algorithm is as follows.

Input: Tasks, VMs, Deadline

Output: Optimize Task-Level Scheduling Plan

1. Initialize scheduling plan (Formula (2));
2. For ITER = 1:maxiteration;
3. Calculate fitness of each scheduling plan (Formula (11));
4. Initialize search velocity of each scheduling plan (Formula (2));
5. Calculate EST, RT and EFT (Formulas (6)–(8));
6. Update current best fitness (Formula (11));
7. For task = 1:tasklist;
8. Select VM;
9. Update search velocity and scheduling plan;
10. Calculate the cost (Formula (10));
11. Update current best fitness (Formula (11));
12. Update the current best solution with chaotic sequences (Formula (2));
13. Decrease the scheduling space and generate new scheduling plan (Formula (3));
14. Construct the new scheduling plan and old top ones;
15. Update the best and its fitness in new scheduling plan (Formulas (2) and (11));
16. End
17. End
18. Return best-possible scheduling plan(s).

For the above algorithm, we first initialize the parameters and the entire scheduling plan (line (1)). The velocity and position of scheduling plans are initialized by the chaotic sequence and adaptive inertia weight factor (lines (3)-(4)). Then the EST, RT, and EFT of scheduling plans are calculated, and the current best fitness is updated (lines (5)-(6)). The scheduling plan chooses VMs for every task and calculates the cost and then updates the current best fitness (lines (8)–(11)). The current best scheduling plan is selected by the chaotic sequence and adaptive inertia weight factor (lines (12)–(15)). When scheduling plan converges to the optimum, the inertia weight factor will decrease. By this way, the scheduling plan will search in local way. In contrast, the inertia weight factor will increase, and the plan will search in global way. By this method, the algorithm decreases the scheduling space and generates new scheduling plan. At last, it returns best-possible scheduling plan(s) with deadline constraint (line (18)).

## 6. Experiments

**Figure 3:**
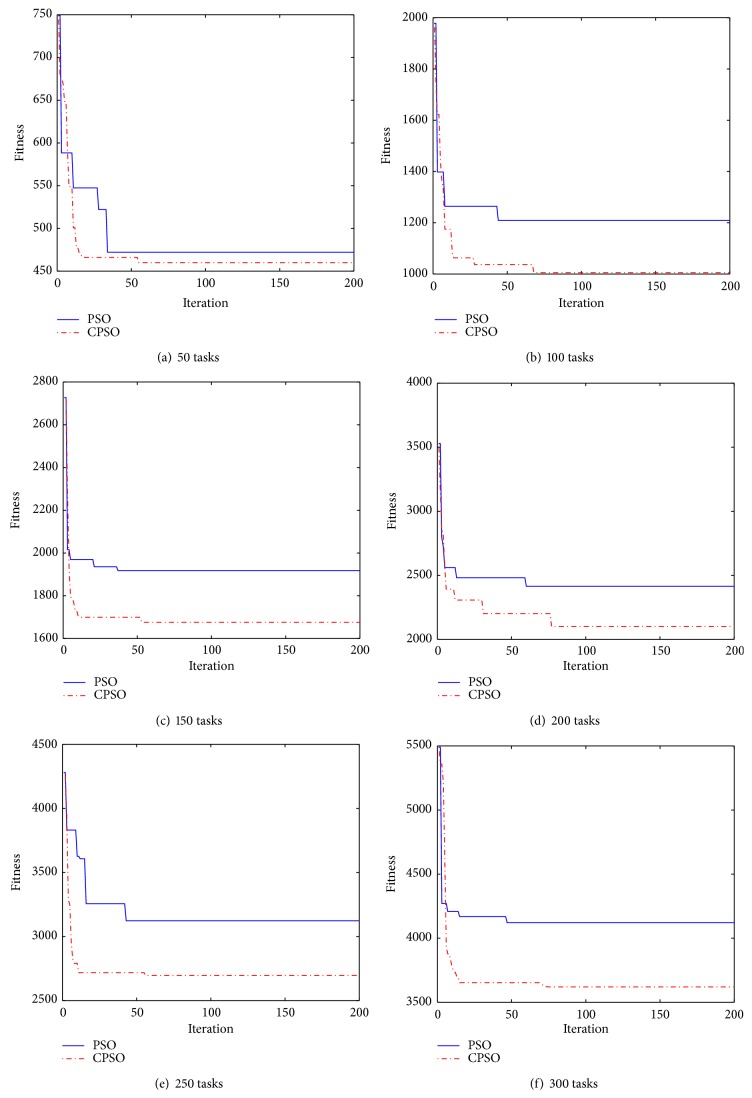
Comparison of fitness between PSO and CPSO.

**Figure 4:**
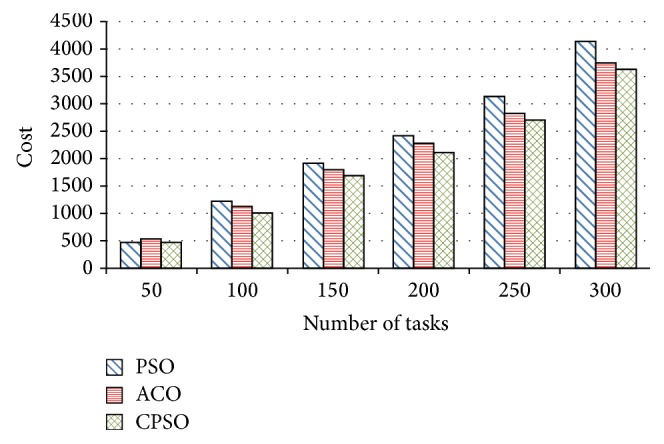
Comparison of cost among ACO, PSO, and CPSO.

**Figure 5:**
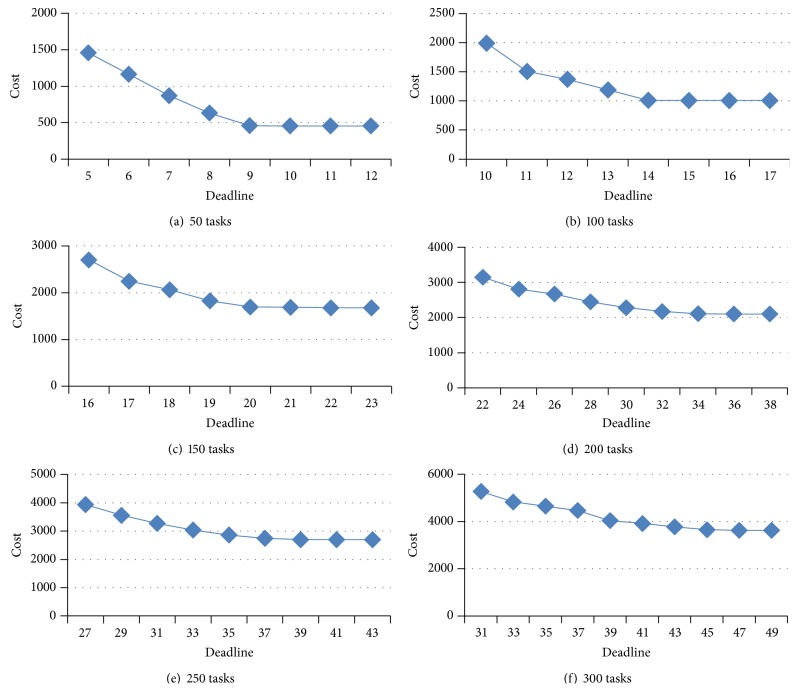
Cost with deadline.

In this section, environment and parameter setting are described. Experimental simulation and analysis are presented in Figures 3–5.

### 6.1. Environment and Setting

The amount of workflow tasks is randomly generated between 50 and 300, and the maximum amount of purchased cloud instances is 10. The structure of workflow topology is generated randomly. The average execution time of each task is random from 10 to 100 basic time units. The amount of VMs is 4 [33]. The type of VM is decided randomly. According to Amazon, the execution speed and price of VMs are shown in Table 3. Every experiment runs 100 times and gets their average value.

In ACO algorithm, the ant amount is 50 and the maximum iteration times are 100. Other parameters are set according to [17]. In PSO and CPSO algorithms, the swarm size is set to 40 for all experiments. The maximum iteration times are 100. The acceleration coefficient are both fixed to 2 [21]. In PSO algorithm the inertia weight factor is fixed to 0.73 [21]. However, it changes according to Formula (3) in CPSO algorithm. And the two factors *w*_min⁡_ and *w*_max⁡_ are set as 0.2 and 1.2, respectively, according to [32].

The deadline_min⁡_ and deadline_max⁡_ are set according to Formula (9). The total cost of the task set is the multiplication of the execution time of tasks and the price of their VMs. Each task set runs 50 times.

### 6.2. Comparison of Fitness

Figure 3 compares the fitness of CPSO-based scheduling with that of the PSO-based scheduling. In Figure 3, we show six experiments with the number of tasks ranging from 50 to 300. The convergence of CPSO-based and PSO-based scheduling is similar. But the fitness of the CPSO-based scheduling is always lower than the PSO-based scheduling. This means that the optimization results of our scheduling is better than the PSO-based scheduling. It is because the chaotic sequences update the current best solution to choose the VMs with the cost to execute tasks. The scheduling plan can reduce the cost of tasks and therefore decrease the total cost of task sets. In Figure 3(a), the two types of fitness are close, because the number of tasks is too small to show obvious difference. In conclusion, chaotic sequences can avoid the premature convergence for our scheduling to find the best-possible cost efficiently.

### 6.3. Comparison of Cost

Figure 4 shows the comparison of cost between ACO, PSO, and CPSO. As shown in Figure 4, the parameters of PSO and CPSO are set the same as in Section 6.1. The cost of the CPSO-based is the lowest and premature convergence can be avoided by our scheduling. The chaotic sequence with high randomness improves solutions diversity, and its regularity assures a good global convergence. Adaptive inertia weight factor controls a proper balancing between global and local exploration to make the scheduling avoid premature convergence.

The cost of scheduling increases with the variation of task amount. When the task amount is 50, the cost of the CPSO-based is 458.9. It is similar to the cost of the ACO-based and the PSO-based scheduling. With the increase of task amount, our cost is always the lowest. In particular, when the amount of tasks is 300, the cost of the PSO-based scheduling is 4120.9, and the cost of the ACO-based scheduling roughly shrinks by 9.4%, namely, 385.8, and the cost of the CPSO-based scheduling is about 12.2% reduction, namely, 501.2. In conclusion, our scheduling can optimize the global best cost efficiently with the CPSO, instead of ACO and PSO.

### 6.4. Cost with Deadline Constraint

In Table 4, deadline_min⁡_ and deadline_max⁡_ of randomly generated tasks are calculated according to Formula (9).

Figure 5 states how the deadline is set and what effect the deadline has on cost. In Figure 5, other parameters are the same as in Section 6.1. In Figure 5(a), the deadline is 4.2, but there is no solution. By experiments, there are some solutions, when the deadline is 5, which is the start value. As the deadlines are 9 and 10, the cost are 458.9 and 455.9. The cost of near-best solution is 458.9 according to Figure 4. While the deadline varies from 5 to 9, the cost gradually decreases. When the deadline is more than 9, the cost becomes stable. The near-best solution can be found while the deadline is 12, which is the end value. For other different task sets, the deadline is changed in the same way according to Table 4.

In conclusion, when deadline is near deadline_min⁡_, there may be no suitable solution. While deadline is between deadline_min⁡_ and deadline_max⁡_, the cost will reduce with the increase of deadline. The reason is that more tasks are allocated into higher performance and more expensive VMs. As deadline is bigger than deadline_max⁡_, the cost will not reduce anymore. The reason is that each task has been scheduled into the highest performance and the most expensive VMs. This experiment can guide us to select a suitable deadline.

Overall, our CPSO-based scheduling can overcome premature convergence and achieve smaller cost than the PSO-based scheduling in Figures 3-4. In Figure 3 the CPSO-based has the lowest fitness, and therefore our scheduling strategy is efficient so as to apply to large-scale task sets for cloud workflow. With consideration of cost requirement of users, the algorithm aims to optimize the cost of whole scheduling. In Figure 4, the CPSO-based scheduling can efficiently reduce the cost. Furthermore, with the increase of available deadline, our scheduling always can achieve a near-best cost in Figure 5. Figure 5 presents that the deadline is set by theoretical and experimental value and the cost reduces while ranging the deadline from start value to end value. Therefore, the cost is constrained by the deadline. It is necessary for cloud workflows to pay for the execution of tasks on VMs to cloud providers. If it is expected to obtain smaller cost for cloud workflows, more deadline is necessary.

## 7. Summary and Future Work

In this paper, a task-level scheduling algorithm based on CPSO is presented. It can optimize the cost of whole scheduling and overcome the premature convergence of PSO algorithm to satisfy the market-oriented characteristic of cloud workflow. A series of experiments using our method and comparing the results with other representative counterparts is conducted. The performance of our scheduling is efficient and the cost is the lowest.

In consideration of the market-oriented scheduling, we only focus on the cost. However, other QoS constraints such as reliability, availability, makespan, and security will be investigated in future. Our strategy is a task-level scheduling which only optimizes the mapping of tasks to VMs. Its upper level for the service-level scheduling, which aims at the optimization of tasks to services, also deserves further research. In experiment setting, the amount of VMs is only fixed to 4. When the amount of VMs increases, more tasks can execute simultaneously and therefore the makespan of task set will decrease. So, experiment with more than 4 VMs will be conducted in the future. Furthermore, the market-oriented scheduling from task-level and service-level will be studied.

## Figures and Tables

**Table 1:** Execution Time (hrs.) of Task on VM.

VM Type	Tasks				
	A	B	C	D	E
Small	3.2	8.0	4.8	1.6	4.8
Medium	2.6	6.5	3.9	1.3	3.9
Large	2.0	5.0	3.0	1.0	3.0

**Table:** (a) PSO Initialization

Search variable	Tasks					ET	Cost
	A	B	C	D	E		
*x*_1_^0^	0.6	0.8	0.4	0.2	0.9	11.1	5.6
*v*_1_^0^	0.2	−0.3	0.1	0.4	−0.2		
Plan_1_^0^	2	3	2	1	3		

*x*_2_^0^	0.8	0.7	0.1	0.3	0.9	10	5.5
*v*_2_^0^	0	0.2	0.5	0.1	−0.2		
Plan_2_^0^	3	3	1	1	3		

*x*_3_^0^	0.3	0.8	0.7	0.5	0.9	20.5	6.0
*v*_3_^0^	0.5	−0.4	−0.3	0	−0.3		
Plan_3_^0^	1	3	3	2	3		

Best_0_	3	3	1	1	3	10	5.5

**Table:** (b) CPSO initialization

Search variable	Tasks					ET	Cost
	A	B	C	D	E		
*x*_1_^0^	0.5	0.9	0.7	0.6	0.8	20.5	6.0
*v*_1_^0^	0.2	−0.3	−0.6	−0.2	−0.6		
Plan_1_^0^	1	3	3	2	3		

*x*_2_^0^	0.3	0.5	0.7	0.6	0.1	15.8	4.3
*v*_2_^0^	0.5	0.1	−0.4	−0.2	0		
Plan_2_^0^	1	2	3	2	1		

*x*_3_^0^	0.2	0.3	0.7	0.5	0.3	17.3	4.5
*v*_3_^0^	0.5	0.1	−0.1	−0.2	0.4		
Plan_3_^0^	1	1	3	2	1		

Best_0_	1	3	3	2	1	15.8	4.3

**Table:** (c) PSO first iteration

Search variable	Tasks					ET	Cost
	A	B	C	D	E		
*x*_1_^1^	0.8	0.5	0.5	0.6	0.7	16.7	4.3
*v*_1_^1^	0.1	0.2	−0.2	−0.3	0.1		
Plan_1_^1^	3	2	2	2	3		

*x*_2_^1^	0.8	0.5	0.6	0.4	0.7	16.7	4.3
*v*_2_^1^	−0.1	0.1	0	0.2	0.2		
Plan_2_^1^	3	2	2	2	3		

*x*_3_^1^	0.8	0.4	0.4	0.5	0.6	18.7	4.7
*v*_3_^1^	0.1	0.2	0.1	−0.1	0.2		
Plan_3_^1^	3	2	2	2	2		

Best_1_	3	2	2	2	3	16.7	4.3

**Table:** (d) CPSO first iteration

Search variable	Tasks					ET	Cost
	A	B	C	D	E		
*x*_1_^1^	0.7	0.6	0.1	0.4	0.2	14.6	3.9
*v*_1_^1^	0.1	0	0	0.2	0		
Plan_1_^1^	3	2	1	2	1		

*x*_2_^1^	0.8	0.6	0.3	0.4	0.1		
*v*_2_^1^	−0.1	0	−0.2	0.2	0	14.6	3.9
Plan_2_^1^	3	2	1	2	1		

*x*_3_^1^	0.7	0.4	0.6	0.4	0.7		
*v*_3_^1^	0.2	0	−0.4	0.2	−0.6	16.7	4.3
Plan_3_^1^	3	2	2	2	3		

Best_1_	3	2	1	2	1	14.6	3.9

**Table:** (e) PSO last iteration

Search variable	Tasks					ET	Cost
	A	B	C	D	E		
*x*_1_^2^	0.9	0.7	0.3	0.3	0.8	16.7	4.3
*v*_1_^2^	−0.1	−0.1	0	0.1	0.2		
Plan_1_^2^	3	2	2	2	3		

*x*_2_^2^	0.7	0.6	0.6	0.6	0.9	16.7	4.3
*v*_2_^2^	0.7	0.1	−0.2	−0.3	0.1		
Plan_2_^2^	3	2	2	2	3		

*x*_3_^2^	0.9	0.6	0.5	0.4	0.8		
*v*_3_^2^	0.1	0.4	−0.2	−0.1	−0.3	16.7	4.3
Plan_3_^2^	3	2	2	2	3		

Best_2_	3	2	2	2	3	16.7	4.3

**Table:** (f) CPSO last iteration

Search variable	Tasks					ET	Cost
	A	B	C	D	E		
*x*_1_^2^	0.8	0.6	0.1	0.6	0.2	14.6	3.9
*v*_1_^2^	−0.1	−0.2	0.1	0	0.2		
Plan_1_^2^	3	2	1	2	1		

*x*_2_^2^	0.7	0.6	0.1	0.6	0.1	14.6	3.9
*v*_2_^2^	0.1	0	0.2	−0.1	0.1		
Plan_2_^2^	3	2	1	2	1		

*x*_3_^2^	0.9	0.4	0.3	0.6	0.1	14.6	3.9
*v*_3_^2^	0.1	0	−0.2	−0.2	0		
Plan_3_^2^	3	2	1	2	1		

Best_2_	3	2	1	2	1	14.6	3.9

**Table 3:** Speed and price of Amazon VMs.

VM type	Speed	Reserved		On-demand
		Per-term ($)	Per-hour ($)	Per-hour ($)
Small	1.00	97.50	0.07	0.12
Medium	1.30	390.00	0.28	0.48
Large	1.60	780.00	0.56	0.96

**Table 4:** Theoretical value of deadline.

Deadline	Tasks					
	50	100	150	200	250	300
deadline_min⁡_	4.2	9.2	15.9	21.4	26.1	30.4
deadline_max⁡_	9.8	20.3	32.0	45.5	62.0	72.1
